# Sporadic Human Infections With *Rickettsia japonica* in Yichang, China, 2021–2023

**DOI:** 10.1155/tbed/4832524

**Published:** 2025-07-25

**Authors:** Yuting Ren, Yale Jiang, Zhoufu Xiang, Qi Cheng, Kehan Chen, Jianchun Ma, Jianwei Dai, Weihao Zhang, Wei Hou, Qiang Liu, Liangjun Chen

**Affiliations:** ^1^State Key Laboratory of Virology and Biosafety, Institute of Medical Virology, Taikang Medical School (School of Basic Medical Sciences), Wuhan University, Wuhan, Hubei, China; ^2^Department of Laboratory Medicine, Zhongnan Hospital of Wuhan University, Wuhan University, Wuhan, Hubei, China; ^3^School of Public Health, Wuhan University, Wuhan, Hubei, China; ^4^Department of Infectious Diseases, The First College of Clinical Medical Science, China Three Gorges University and Yichang Central People's Hospital, Yichang, Hubei, China

**Keywords:** clinical features, epidemiological characteristics, Japanese spotted fever (JSF), *Rickettsia japonica*, spotted fever group rickettsiosis

## Abstract

Japanese spotted fever (JSF) is an easily neglected infectious disease, where misdiagnosis and delayed treatment significantly contribute to poor prognoses in affected patients. Our prospective observational study (2021–2023) systematically characterized 56 JSF cases in Yichang through tripartite analysis encompassing epidemiological distributions, clinical phenotyping, and phylogenetic relationship analysis of *Rickettsia japonica* (*R. japonica*). Our study delineated distinct clinical presentations of JSF, and identified five laboratory indexes demonstrating significant associations with disease severity. Notably, thrombocytopenia (platelet deficiency) and procalcitonin (PCT) levels emerged as critical indicators, with PCT as a well-characterized inflammatory mediator showing particular prognostic value for anticipating severe complications in rickettsial infections, consistent with prior pathophysiological research. Phylogenetic analysis revealed that *R. japonica* strains distributed in Yichang City exhibited extremely low genomic diversity. Further, the *R. japonica* strains isolated in our study exhibited a high degree of homology with *R. japonica* isolation within the borders of China. In summary, our research identified several factors that indicate a high risk of poor outcomes in *R. japonica* infections. Additionally, we observed highly similar phylogenetic relationships among *R. japonica* strains, which have important implications for disease prevention, control, and clinical diagnosis.

## 1. Introduction

Japanese spotted fever (JSF) is a severe zoonosis characterized by acute febrile rash and transmitted by ticks [1]. *R. japonica* has been identified as the pathogenic agent of JSF, which has been firstly reported in Tokushima Prefecture, Japan, in 1984 [2]. Since then, JSF has also been prevalent in multiple countries across Asia, including South Korea, the Philippines, and Thailand [3–5]. In recent years, sporadic cases of JSF have emerged in various regions of China, such as Zhejiang Province, Anhui Province, Henan Province, and Hubei Province [6–9]. These reports collectively indicate the increasing spread and global recognition of JSF as a significant public health concern.

The major clinical manifestations of JSF include fever, rash, tick bite eschars, and malaise [10, 11]. In severe cases, JSF can result in pathological damage to various organs, including lung, central nervous system, digestive system, kidney, and heart [12]. The main laboratory findings for JSF include leukocytosis or leukopenia, thrombocytopenia, decreased eosinophils, elevated C-reactive protein (CRP), and raised liver enzymes [11, 13]. Although the overall prognosis for JSF was promising, there have been reports indicating a risk of severe complications, including cases leading to death. *R. japonica* infection can give rise to purpura fulminans outbreaks or complication, such as disseminated intravascular coagulation which is highly life-threatening [14, 15].

JSF demonstrates a higher incidence during the spring and summer months, often associated with tick bites and recent instances of camping or occupational exposure. The pathophysiological progression involves the transmission of the pathogen to the human body through bites inflicted by ticks acting as carriers of the infectious agent. According to previous study, *R. japonica* has been identified in ticks belonging to three genera (*Haemaphysalis, Dermacentor*, and *Ixodes*) and a total of eight species [16].

In this study, our objective was to assess the occurrence of JSF in China using hospital-based surveillance, and investigate the epidemiological characteristics, clinical manifestations, and laboratory results of JSF patients. Furthermore, the findings of the study could serve as a valuable foundation for future research aimed at formulating effective prevention strategies and improving clinical diagnosis for this disease.

## 2. Materials and Methods

### 2.1. Ethics Statement

The study received formal approval from the Medical Ethics Committee of Yichang Central People's Hospital (No. 2021-106-01), in full compliance with China's medical research regulatory requirements.

### 2.2. Study Population

To investigate the progression and clinical features of patients infected with *R. japonica*, a single-center retrospective observational study was carried out from September 2021 to September 2023 at Yichang Central Hospital. The study included a cohort of 56 patients who had been clinically diagnosed as *Rickettsia* infection and possessed complete clinical records. The diagnosis of JSF was based on a combination of clinical symptoms, epidemiological history, and molecular detection. Initially, patients presenting with fever, rash, and eschar, along with a history of exposure to endemic areas, were suspected of rickettsial infection. All the patients were diagnosed with *R. japonica* bloodstream infection by metagenomic next-generation sequencing (mNGS). Among the 56 patients initially screened, 53 patients had adequate residual whole blood samples available for subsequent PCR confirmation. Due to the low pathogen load in peripheral blood, we were only able to obtain sequence information for both the citrate synthase gene (*gltA)* and outer membrane protein B-encoding gene (*ompB)* gene in 12 patients, which were subsequently used for genetic evolutionary analysis.

### 2.3. mNGS

The mNGS workflow included the following steps:

Nucleic acid processing: plasma-derived total nucleic acids were isolated using the MatriDx automated system (Hangzhou, China), with integrity verified by capillary electrophoresis.

Library construction: enzymatic fragmentation (Covaris) was optimized to generate 150–300 bp inserts, followed by end-repair (NEBNext Ultra II), 3′-adenylation (KAPA HyperPrep), and dual-index adapter ligation (IDT for Illumina). Library quality was assessed by Qubit fluorometry and Bioanalyzer profiling.

Sequencing: normalized libraries were pooled at equimolar ratios and sequenced on an Illumina NextSeq500 (High Output Kit v2.5) to yield ~ 20M single-end 50 bp reads per sample (Q30 >85%).

Bioinformatic analysis: reads aligning to hg38 (Bowtie2, stringent mode) were discarded. Nonhost reads were queried against a curated database integrating NCBI nt (v2025), RefSeq pathogens, and clinical metagenomics signatures (Kraken2/Bracken). Extraction blanks and PCR negatives were processed in parallel to establish background thresholds (<0.001% of total reads).

The mNGS data generated in this study have been deposited on the NCBI under the accession number PRJNA1254936. These data are publicly available for validation and further analysis.

### 2.4. Participants and Grouping Standard

Consistent with the Third International Consensus Definitions for Sepsis and Septic Shock (Sepsis-3), severe cases were defined as patients meeting both criteria: requiring continuous vasoactive agents (norepinephrine ≥0.1 μg/kg/min) to maintain mean arterial pressure (MAP) ≥65 mmHg after ≥30 mL/kg crystalloid resuscitation; serum lactate >2 mmol/L despite adequate volume resuscitation. Patients not fulfilling these hemodynamic and metabolic derangement thresholds were designated as mild cases [17].

In this study, laboratory findings collected within the first 24 h of admission were included for analysis. Therefore, some data may be incomplete due to certain laboratory tests not being conducted upon admission. The retrospective observational nature of this study was reviewed and certified as exempt from ethical approval by the ethics review committee of our hospital. To ensure patient privacy, identifiable information in figures was concealed, and informed consent was obtained from all participants involved.

Organ failure was stratified according to the sequential organ failure assessment (SOFA) criteria, requiring concurrent dysfunction across ≥2 organ systems sustained for >24 h. Acute hypoxemic respiratory failure (PaO_2_/FiO_2_ ≤200 mmHg on PEEP ≥5 cmH_2_O) per Berlin ARDS criteria for respiratory; refractory hypotension (MAP <65 mmHg despite norepinephrine ≥0.1 μg/kg/min) with serum lactate >2 mmol/L, aligned with surviving sepsis campaign 2021 guidelines; stage 2 acute kidney injury (KDIGO criteria) defined as: serum creatinine ≥2.0 mg/dL (baseline-adjusted) OR renal replacement therapy initiation within 48 h. Inclusion required objective evidence of ≥2 concurrent organ failures persisting beyond initial resuscitation phase (first 6 h) [18].

### 2.5. DNA Extraction, PCR, Sequencing and Phylogenetic Analysis

Total of 53 whole blood samples from these JSF patients were collected and Rickettsiales genomic DNA was extracted using the DNeasy Blood and Tissue Kit (Qiagen, Japan) according to the manufacturer's instructions. Detection of Rickettsiales DNA was performed by nested PCR, which amplified a partial region of the *gltA* gene (gltA-out1 [5′-GATTGCTTTACTTACGACCC-3′], gltA-out2 [5′-TGCATTTCTTTCCATTGTGC-3′], gltA-in1 [5′-TATAGACGGTGATAAAGGAATC-3′], gltA-in2 [5′-CAGAACTACCGATTTCTTTAAGC-3′]) and *ompB* gene (ompB-out1 [5′-ATATGCAGGTATCGGTACT-3′], ompB-out2 [5′-CCATATACCGTAAGCTACAT-3′], ompB-in1 [5′-G CAGGTATCGGTACTATAAAC-3′], and ompB-in2 [5′-AATTTACGAAACGATTACTTCCGG-3′]).

Genomic DNA sequences of these two bacterial genes obtained in this study were aligned with GenBank reference sequences using the ClustalW algorithm (default parameters) in MEGA software, version 7.0 [19]. Phylogenetic reconstruction was subsequently performed using datasets of varying sizes: (i) a 400 bp *gltA* gene (*N* = 49 sequences) and (ii) a 700 bp *ompB* gene (*N* = 39). ModelFinder module in IQ-TREE was employed to determine the optimal substitution model for each sequence alignment [20]. The phylogenetic reconstruction process was conducted using a maximum likelihood (ML) framework implemented through the General Time Reversible substitution model with parameter optimization for invariant sites and a four-category discrete gamma distribution (GTR + I+G4), which was determined to be the most appropriate evolutionary model through model selection procedures. The tree topology optimization employed a heuristic search strategy featuring nearest-neighbor-interchange (NNI) branch swapping algorithms. Statistical support for nodal robustness was assessed through nonparametric bootstrapping with 1000 pseudoreplicates executed in MEGA software. For comparative evolutionary interpretation, all resultant cladograms were subjected to midpoint rooting normalization prior to topological evaluation. All obtained sequences have been deposited in GenBank under accession numbers PQ141052 to PQ141063.

### 2.6. Statistical Analysis

Statistical analyses were executed utilizing IBM SPSS Statistics (version 27.0; IBM Corp., Armonk, NY, USA) with rigorous adherence to parametric assumptions. Quantitative measures were characterized through parametric descriptors (mean ± standard deviation) or nonparametric representation. Categorical parameters were quantified using frequency distributions with proportional percentages. Intergroup comparisons between severe and mild clinical cohorts employed hypothesis-driven analytical frameworks: Pearson's *χ*^2^ test or Fisher's exact test for categorical variables, independent Student's *t*-test or analysis of variance (ANOVA) for normally distributed continuous variables, supplemented by Mann–Whitney *U* or Kruskal–Wallis tests for nonGaussian distributions. Furthermore, bivariate association patterns were systematically investigated through univariate logistic regression modeling, with severity stratification serving as the dichotomous outcome variable, reporting odds ratios (ORs) with 95% confidence intervals to quantify effect magnitudes.

## 3. Results

### 3.1. Demographic Characteristics of JSF Patients From Yichang City, Hubei Province, 2021–2023

**Figure 1:**
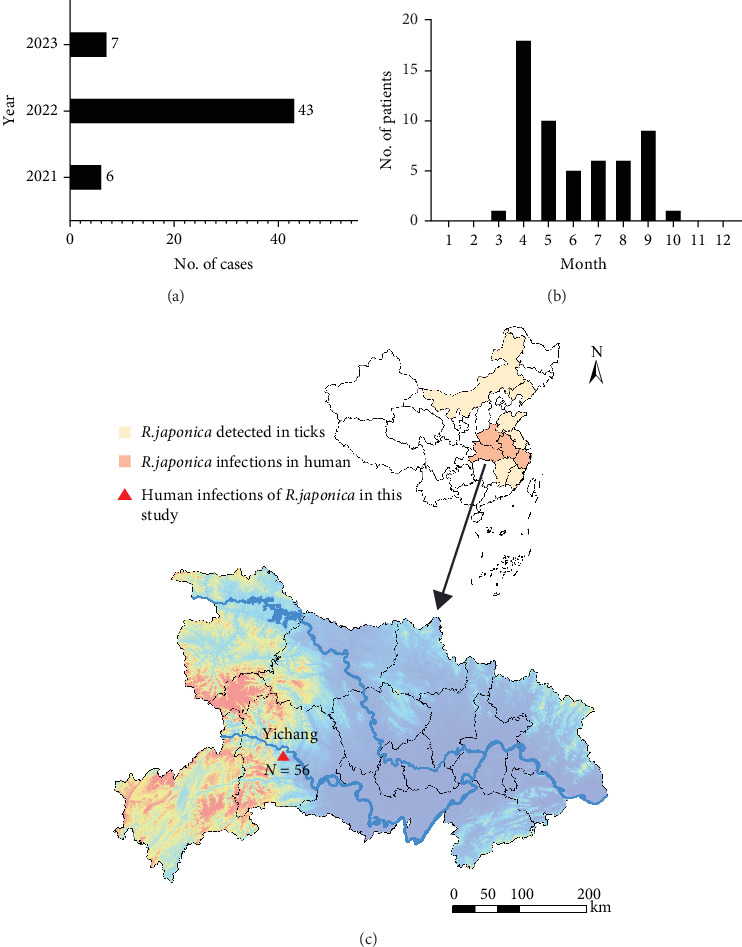
Epidemiological data of JSF patients in Yichang, Hubei Province from 2021 to 2023. (A) Clinical confirmed cases of JSF in Yichang, Hubei Province from 2021 to 2023. (B) Month distribution of clinical confirmed JSF cases. (C) Geographical distribution of *Rickettsia japonica* in China. Provinces where *R. japonica* has not been detected are displayed in white, if detected in ticks, marked in yellow, if detected in human-related samples, marked in orange. Spatio-epidemiological visualization was implemented through cartographic symbology where cardinal red triangular feature demarcated geolocated residences of clinically confirmed JSF cases. The cartographic workflow comprised: (1) geodatabase construction and kernel density estimation in ArcGIS 10.2 (Environmental Systems Research Institute, USA) employing WGS1984 coordinate system; (2) topographic refinement through curve optimization in Adobe Illustrator CC 2018 (Adobe, San Jose, CA, USA). Basemap hydrographic layers were sourced from China's National Geospatial Data Infrastructure (NGDI) via the Tianditu portal (https://www.tianditu.gov.cn;GS(2020)1821 geodetic standard).

A retrospective cohort of 56 clinically diagnosed *Rickettsia japonica* infection cases receiving inpatient care at Yichang Central People's Hospital during September 2021 to September 2023 surveillance window was systematically enrolled (Figure 1A). The JSF cases were all distributed from March to October, indicating the prevalence of JSF from spring to autumn, which has significant temporal congruence with ixodid tick activity peaks (Figure 1B). According to current studies, among the 34 provinces and special administrative regions in China, 10 (29.4%) provinces tested nucleic acid positive of *R. japonica*, and four provinces had reported *R. japonica* infections in human [21]. All of 56 JSF patients in this study resided in Yichang City, Hubei Province, indicating the small-scale outbreak of *R. japonica* infection there, from 2021 to 2023.

### 3.2. Clinical Characteristics of JSF Patients From Yichang City, Hubei Province, 2021–2023

Among the 56 case-patients (33 females and 23 males), median age of case-patients was 60 years, ranging from 38 to 80 years. Seven patients required admission to the intensive care unit (ICU), but all patients have recovered after treatment with doxycycline. Importantly, there were no reported deaths among the case-patients in our study, probably because patients in this study had few underlying conditions or critically ill patients were transferred to higher-level hospitals for specialized care. Almost half of these patients (25, 46.3%) had a history of tick bites, while 20.4% of them (11 patients) were unaware of being bitten by ticks (Table 1).

The most common clinical manifestations observed in the 56 JSF patients were fever (100.0%), rash (98.2%), and fatigue (89.3%), ranked by descending prevalence rates (Supporting Information 1: Table S1). To identify risk factors for severe disease, clinical symptoms and laboratory parameters were analyzed using logistic regression. Interestingly, thrombocytopenia (OR 0.111, 95% CI 0.013–0.935; *p*=0.043) and multiorgan failure (OR 0.113, 95% CI 0.018–0.712; *p*=0.020) were found to exert measurable impacts in severe JSF patients. In conclusion, our study characterized the clinical data of 56 JSF patients from Yichang City, Hubei Province, with an adequate sample size covering the period from 2021 to 2023.

**Figure 2:**
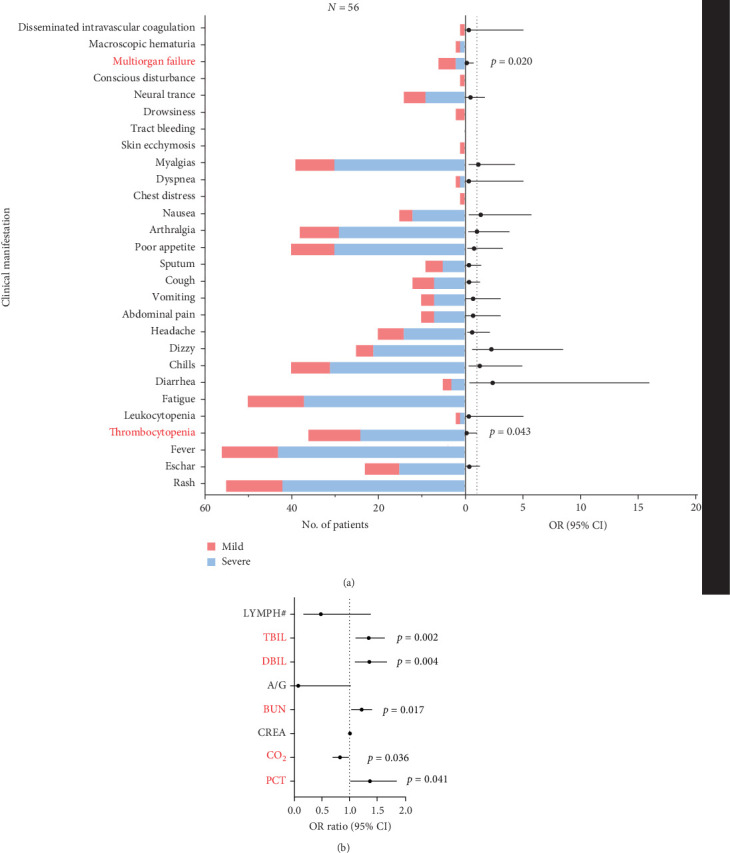
The proportion/level and OR for severe case of JSF patients by clinical manifestations (A) and laboratory indexes (B). The black points are the ORs for severe case and the black error bars are the 95% CIs. The dotted line indicates an adjusted OR of 1. OR,odds ratio.

The most common laboratory indexes are outlined in Supporting Information 2: Table S2. By comparing the mean values of these laboratory indicators with the normal range, we observed that JSF patients had elevated levels of neutrophil ratio (NEUT%), d-dimer (DD), creatine kinase (CK), lactate dehydrogenase (LDH), aspartate aminotransferase (AST), glutamic pyruvic transaminase (ALT), and inflammatory indicator including procalcitonin (PCT), interleukin- 6 (IL-6), CRP, and serum amyloid A (SAA), while carbon dioxide (CO_2)_ was decreased (Supporting Information 2: Table S2). To further delineate the relationship between laboratory variables and disease severity, we divided the patients into two groups: mild cases and severe cases, based on the development of septic shock. Among these groups, a total of eight laboratory variables showed significant differences, including lymphocyte count (LYMPH#) (*p*=0.028), albumin/globulin (ALB/GLB) (*p*=0.046), blood urea nitrogen (BUN) (*p*=0.006), creatinine (CREA) (*p*=0.028), CO_2_ (*p*=0.006), PCT (*p*=0.014), total bilirubin (TBIL) (*p*=0.012), direct bilirubin (DBIL) (*p*=0.019). Furthermore, we conducted multivariable logistic regression framework to assess the correlation between laboratory variables and clinical severity. The results showed that TBIL (OR 1.350, 95% CI 1.113–1.639; *p*=0.002), DBIL (OR 1.360, 95% CI 1.100–1.680; *p*=0.004), BUN (OR = 1.211, 95% CI 1.034–1.417; *p*=0.017), CO_2_ (OR 0.828, 95% CI 0.695–0.988; *p*=0.036) and one inflammatory biomarker, PCT (OR 1.372, 95% CI 1.013–1.857; *p*=0.041) were significantly correlated with clinical severity (Figure 2).

### 3.3. Phylogenetic Analysis of *R. japonica* Strains

DNA was extracted from peripheral blood samples collected during the acute phase of illness. The samples were then screened using a nested PCR assay that targeted both *gltA* and *ompB*. According to previous studies [22, 23], all the 56 patients indicated nucleic acid positive for *R. japonica* using mNGS. However, in some samples, the load of *R. japonica* was too low to be amplified by nested PCR targeting the fragments of gene *gltA* and gene *ompB*. As a result, we detected nucleic acid positive of *R. japonica* in 12 patients resided in Yichang City, Hubei Province.

**Figure 3:**
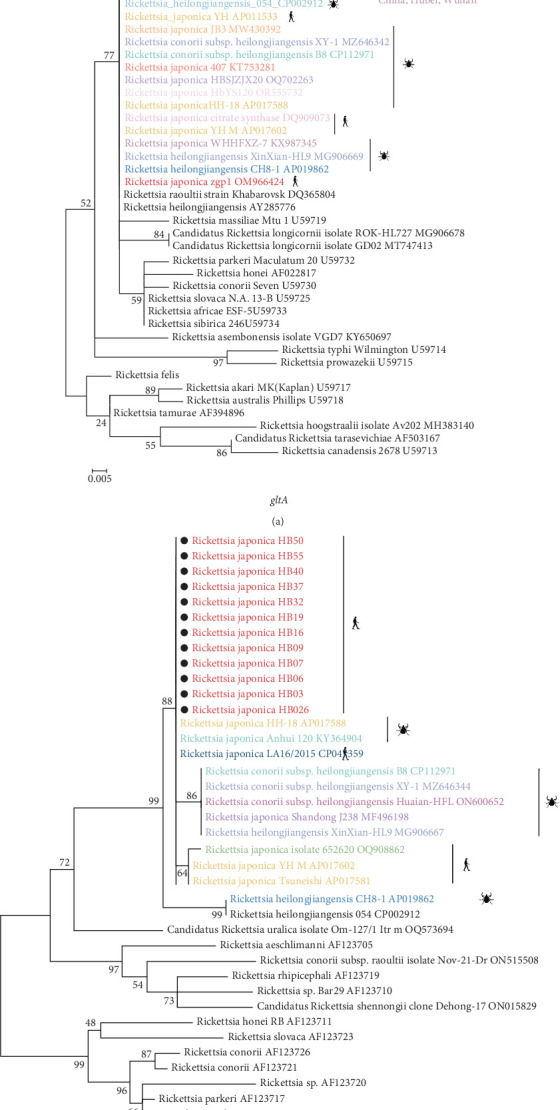
Phylogenetic trees based on partial sequences of *gltA* (A) and *ompB* (B) gene. Phylogenetic reconstructions were subjected to midpoint rooting methodology to optimize topological visualization Bootstrap values are annotated exclusively for clades demonstrating statistically significant value. The accompanying scale indicator corresponds to nucleotide substitution rates per sequence position. The analysis was performed using the *gltA* region between positions 518 and 952 (434 bp), *ompB* region between positions 1420 and 2110 (690 bp).

The phylogenetic analysis of the *gltA* gene and *ompB* gene revealed a high degree of similarity between the *R. japonica* strains in our samples and Japanese isolates, with homology ranging from 99.8% to 100% (Figure 3). Furthermore, our samples clustered together with *R. japonica* strains identified in ticks, such as the *R. japonica* strain HH-18, from various locations. Notably, our samples showed complete homology (100%) with *R. japonica* isolates from Zhejiang Province, China in 2015. In summary, our study demonstrated a close genetic relationship between *R. japonica* strains in Yichang City, Hubei Province, and those found in other regions of China.

## 4. Discussion

*R. japonica*, a member of the spotted fever group rickettsiae (SFGR), can cause JSF in humans. This study is the first long-term monitoring report of JSF in Yichang City, Hubei Province. We surveyed and recruited 56 JSF patients at a single center in Yichang City, and conducted timely and systematic research on assessing the molecular epidemiological characteristics, clinical features and *R. japonica* diversity.

The first documented clinical case of *R. japonica* infection in China was reported in Anhui Province in November 2013 [7]. Subsequently, from 2014 to 2017, there were 14 cases of JSF in Xinyang City, Henan Province and 16 cases in Zhejiang Province [6, 8]. Nevertheless, previous studies suggested that *R. japonica* may be more prevalent in China than previously believed. Yichang City is also recognized as a high-risk area for tick-borne severe fever with thrombocytopenia syndrome (SFTS) [24]. Hence, it is crucial to conduct comprehensive and accurate diagnoses of both SFTS and SFGR diseases, and healthcare professionals should maintain a high level of vigilant regarding the potential for SFTSV and SFGR infections.

Common clinical manifestations of JSF patients from Japan include fever, chills, headache, as well as rash and tick bite eschars [11]. Similarly, in our study, we also observed fever, chills, and rash, but fewer headaches and eschar symptoms were witnessed. Nevertheless, we found a high prevalence of fatigue symptoms among our JSF cases, which was consistent with the JSF patients in Xinyang, Henan Province [8]. The differences in clinical manifestations between our study and previous reports may be attributed to various factors, including geographical variations, differences in patient populations, and individual variability in disease presentation.

Furthermore, our study identified a clinical manifestation, thrombocytopenia that showed a significant correlation with the severity of JSF. From physiological perspective, *Rickettsiae* bacteria primarily invade vascular endothelial cells, causing damage to the microvasculature. This damage triggers platelet consumption, resulting in thrombocytopenia [25]. Our findings support the previous research that emphasized the degree of thrombocytopenia serves as a crucial prognostic factor for Rickettsial diseases [26]. In addition, studies have shown that if Rickettsial infections are not promptly treated, severe complications such as fulminant purpura, diffuse intravascular coagulation complications, and multiple organ failure may occur [15, 27]. Le Van et al. [25] demonstrated that PCT levels can be used in combination with other clinical variables as a primary inflammatory marker to predict severe complications of Rickettsial disease. In this study, PCT was shown to be significantly associated with JSF disease severity, supporting its use as a biomarker for disease progression in *R. japonica* infection. These clinical observations in this study not only expand our understanding of the disease spectrum associated with *R. japonica* infection but also emphasize the significance of recognizing and monitoring thrombocytopenia and PCT levels in JSF patients.

There are some limitations in our study. First, we collected samples exclusively from a single hospital, which may have introduced selection bias. Critically ill patients were transferred to superior hospitals, and this limited our ability to capture comprehensive data on fatalities. Second, due to the constraints associated with retrospective analysis, only medical records containing complete information were included in the study. Therefore, all the results of this study may not be fully representative of the clinical manifestations of Rickettsial disease. Finally, the relatively small sample size may have hindered the detection of certain significant differences.

Akter et al. [28] conducted a comparison of the complete sequences of 31 *R. japonica* isolates obtained from different sources in Japan spanning the last 30 years, and revealed an exceptionally low level of genomic diversity, with only 34 single nucleotide polymorphisms detected among 27 primary lineages of the three tick isolates, encompassing all clinical isolates. Similar to the previous results, *R. japonica* strains detected in our study clustered together on the same branch of the phylogenetic tree, manifested the extremely low level of genetic variability. In addition, genetic evolution analysis manifested a significant similarity between our strains and the *R. japonica* strain LA16/2015, which was isolated in Zhejiang Province, within the territory of China. This suggests that a genetically distinct subtype of *R. japonica* has been emerged in different regions of China.

Sporadic JSF infections have occurred in central mountainous areas and southeast coastal cities in China. While it is made strides in this natural epidemic disease, there still remains a lack of comprehensive nationwide data on the distribution of JSF and its vectors. Therefore, it is imperative to enhance the national epidemiological investigation of JSF and intensify monitoring of its hosts and vectors. This will provide valuable insights for disease prevention and control as well as clinical diagnosis.

## Figures and Tables

**Table 1:** Demographic characteristics and laboratory variables in JSF patients.

Groups	Total (*N* = 56)	Mild (*N* = 43)	Severe (*N* = 13)	*p*-Value
Sex (no. [%])				
Male	23 (41.1)	17 (39.5)	6 (46.2)	0.753
Female	33 (58.9)	26 (60.5)	7 (53.8)	
Age (year)				
Median (range)	60.29 (38–80)	59.6 (39–80)	62.54 (38–73)	0.360
Admitted to ICU (no. [%])				
Yes	7 (12.7)	2 (4.8)	6 (38.5)	0.007
No	48 (87.3)	40 (95.2)	8 (61.5)	
Clinical outcome (no. [%])				
Survive	55 (100.0)	42 (100.0)	13 (100.0)	—
Died	0 (0.0)	0 (0.0)	0 (0.0)	
History of tick bite (no. [%])				
Yes	25 (46.3)	19 (46.3)	6 (46.2)	0.493
No	18 (33.3)	15 (36.6)	3 (23.1)	
Unknown	11 (20.4)	7 (17.1)	4 (30.8)	
Days from symptom onset				
Median (range)	7.38 (1–20)	7.40 (2–20)	7.31 (1–19)	0.944
Laboratory indicators (mean ± SD)				
LYMPH# (×10^9^/L)	1.10 ± 1.02	1.20 ± 1.11	0.72 ± 0.39	0.028
TBIL (μmol/L)	11.84 ± 7.35	9.49 ± 3.03	21.00 ± 11.50	0.012
DBIL (μmol/L)	5.62 ± 4.65	4.27 ± 2.18	11.05 ± 7.51	0.019
A/G	1.21 ± 0.30	1.26 ± 0.31	1.07 ± 0.22	0.046
*⁣*^*∗*^BUN (mmol/L)	7.13 ± 4.28	6.30 ± 3.42	10.34 ± 5.82	0.006
*⁣*^*∗*^CREA (μmol/L)	99.1 ± 85.62	85.11 ± 58.24	144.28 ± 136.03	0.028
CO_2_ (mmol/L)	23.58 ± 4.45	24.28 ± 4.26	20.80 ± 4.31	0.026
PCT (ng/mL)	1.73 ± 2.33	1.31 ± 1.80	3.23 ± 3.32	0.014

*Note:* Statistical modeling of categorical covariates was implemented through contingency table analysis, with dichotomous clinical parameters (sex, ICU admission status, and tick exposure history) analyzed using Pearson's *χ*^2^ test, supplemented by Fisher's exact test for expected frequencies <5. Continuous variables (age at onset, symptom-to-admission latency, and hematological indices) underwent parametric analysis via independent two-sample *t*-test. NonGaussian distributed laboratory markers (denoted by asterisks) were subjected to Kruskal–Wallis *H*-test. All inferential analyses adopted two-tailed testing protocols, with statistical significance threshold set at *p*=0.05 per contemporary biostatistical guidelines.

## Data Availability

The data that support the findings of this study are available from the corresponding author upon reasonable request.

## Supporting Information

Supporting Information 1 Table S1: Presence of clinical manifestations during hospitalization among JSF patients with different disease severity.

Supporting Information 2 Table S2: Laboratory index during hospitalization among JSF patients with different disease severity.
